# Maternal Bacterial Engraftment in Multiple Body Sites of Cesarean Section Born Neonates after Vaginal Seeding—a Randomized Controlled Trial

**DOI:** 10.1128/mbio.00491-23

**Published:** 2023-04-19

**Authors:** Noel T. Mueller, Moira K. Differding, Haipeng Sun, Jincheng Wang, Shira Levy, Varsha Deopujari, Lawrence J. Appel, Martin J. Blaser, Tanima Kundu, Ankit A. Shah, Maria Gloria Dominguez Bello, Suchitra K. Hourigan

**Affiliations:** a Department of Epidemiology, Johns Hopkins University Bloomberg School of Public Health, Baltimore, Maryland, USA; b Welch Center for Prevention, Epidemiology and Clinical Research, Baltimore, Maryland, USA; c Department of Biochemistry and Microbiology, Rutgers University, New Brunswick, New Jersey, USA; d Clinical Microbiome Unit (CMU), Laboratory of Host Immunity and Microbiome, National Institute of Allergy and Infectious Diseases, National Institutes of Health, Bethesda, Maryland, USA; e Inova Children’s Hospital, Inova Health System, Falls Church, Virginia, USA; f Center for Advanced Biotechnology and Medicine, Rutgers University, New Brunswick, New Jersey, USA; g Inova Women’s Hospital, Inova Health System, Falls Church, Virginia, USA; h Department of Anthropology, Rutgers University, New Brunswick, New Jersey, USA; i Institute for Food, Nutrition and Health, Rutgers University, New Brunswick, New Jersey, USA; j Canadian Institute for Advanced Research (CIFAR), Toronto, Ontario, Canada; University of Maryland, School of Medicine

**Keywords:** vaginal seeding, vaginal microbiome transfer, Cesarean section, microbiome, microbiota, neonate, randomized controlled trial

## Abstract

Children delivered by elective, prelabor Cesarean section (C-section) are not exposed to the birth canal microbiota and, in relation to vaginally delivered children, show altered microbiota development. Perturbed microbial colonization during critical early-life windows of development alters metabolic and immune programming and is associated with an increased risk of immune and metabolic diseases. In nonrandomized studies, vaginal seeding of C-section-born neonates partially restores their microbiota colonization to that of their vaginally delivered counterparts, but without randomization, confounding factors cannot be excluded. In a double-blind, randomized, placebo-controlled trial, we determined the effect of vaginal seeding versus placebo seeding (control arm) on the skin and stool microbiota of elective, prelabor C-section-born neonates (*n* = 20) at 1 day and 1 month after birth. We also examined whether there were between-arm differences in engraftment of maternal microbes in the neonatal microbiota. In relation to the control arm, vaginal seeding increased mother-to-neonate microbiota transmission and caused compositional changes and a reduction in alpha diversity (Shannon Index) of the skin and stool microbiota. The neonatal skin and stool microbiota alpha diversity when maternal vaginal microbiota is provided is intriguing and highlights the need of larger randomized studies to determine the ecological mechanisms and effects of vaginal seeding on clinical outcomes.

## INTRODUCTION

The microbes that first colonize a neonate at birth play a key role in metabolic programming and immune system development (1–3). Perturbing the timing and order of the sequential microbiota colonization has been shown to exert long-lasting metabolic, immune, and inflammatory consequences (4, 5).

Cesarean section (C-section)—31.8% of all births in the United States in 2020 (6)—is often necessary and lifesaving but is also associated with perturbed microbial colonization of the neonate (7). Experiments in mice show that altering early-life microbiota colonization causes immune and metabolic phenotypic alterations (5). Consistently, epidemiological evidence supports that C-section delivery is associated with an increased risk for several inflammatory and metabolic conditions. At least three meta-analyses of observational studies have concluded that C-section delivery is associated with a higher offspring risk of developing overweight and obesity, even after controlling for potential confounding factors (8–10). In addition, a large longitudinal study found that among siblings born to the same mother, vaginal birth after C-section decreases the offspring risk of developing obesity in childhood, adolescence, and adulthood by 31% (11). There is also evidence that C-section increases the risk of other inflammatory conditions, including atopy (12). The relationship between C-section and these health outcomes may in part be mediated by the microbiome, as suggested by a recent large longitudinal birth cohort study with repeated measures and by experiments in murine models showing a transfer of these adverse disease phenotypes with microbiota transplantation (5, 13).

Neonates delivered by C-section bypass the vaginal canal and therefore miss out on the exposure to the first live microbes and pioneer microbial colonizers that babies receive during vaginal birth, namely, the vaginal-perineal maternal microbes. Numerous studies confirm substantial differences in the microbiota acquisition and maturation in neonates delivered by C-section compared with vaginal delivery (14–20). Swabbing C-section-delivered neonates immediately after birth with mother’s vaginal fluids, coined “vaginal seeding,” is a means to transfer the mother’s vaginal microbiota to the newborn. It is hypothesized that vaginal seeding restores microbes that would have colonized the newborn during vaginal delivery, with the prospect that it may reduce the risk of C-section-associated adverse health outcomes (21). Observational data support the hypothesis that vaginal seeding may partially restore the microbiota of C-section delivered infants (22, 23). In a small pilot study of 4 neonates who underwent vaginal seeding, gut, oral, and skin bacterial communities were enriched by vaginal bacteria in the first month of life, comparable to levels in vaginally delivered neonates (22). A follow-up, larger observational study showed infants who received vaginal seeding had microbiome trajectories that aligned more closely with vaginally delivered infants than with C-section-delivered infants who did not receive vaginal seeding over the first year of life, with a marked enrichment of gut *Bacteroides* (23). However, without randomization, confounding factors explaining part of the observed effect cannot be ruled out.

C-section is correlated with other factors that could perturb the microbiota of the baby, including perinatal antibiotics, shorter gestational time, and lack of labor. No previous study has determined in a randomized, placebo-controlled trial, the effect of transplanting microbes via vaginal seeding to neonates that are extracted from the uterus into the air of operating rooms. In this double-blind, randomized, placebo-controlled study, we assess causation in the relationship between vaginal seeding of C-section-delivered babies and changes in the microbiome. We compare the fecal and skin microbiota of neonates randomly assigned to being seeded by their mother’s vaginal microbiome versus placebo seeding (control). We further assessed engraftment of maternal microbes in the infant microbiota by comparing the proportion of microbes shared between mothers and their children.

## RESULTS

### Subjects.

Twenty mother-child dyads were randomized to either vaginal seeding (*n* = 10) or placebo seeding with sterile saline (control group, *n* = 10). The mean age of the mothers was 33.4 years (range, 27 to 41 years) and the mean maternal prepregnancy body mass index (BMI) was 25.6 kg/m^2^ (range, 18.5 to 33.7 kg/m^2^). Of the 20 neonates, 60% were male, mean birth weight was 3.21 kg (range, 2.22 to 3.95 kg), 18/20 received exclusive breast milk as their first feed, and 12/20 were exclusively breastfed at 7 days. See Table S1 in the supplemental material for detailed maternal and neonatal demographic and clinical characteristics. Baseline characteristics were similar between treatment arms (Table S1).

10.1128/mbio.00491-23.1 TABLE S1 Baseline characteristics of randomized infants and mothers. Download Table S1, PDF file, 0.02 MB. Copyright © 2023 Mueller et al. 2023 Mueller et al. https://creativecommons.org/licenses/by/4.0/ This content is distributed under the terms of the Creative Commons Attribution 4.0 International license.

Vaginal seeding was performed immediately after delivery and before skin-to-skin contact with their mother, following the procedure performed in our previous observational studies of vaginal seeding (22, 23). In the vaginal-seeding group, the mean time from the removal of the vaginal gauze to swabbing the infant was 46 min (range, 31 to 62 min). There were no vaginal seeding-related adverse events in any of the infants of the study.

### The maternal microbiota.

**FIG 1:**
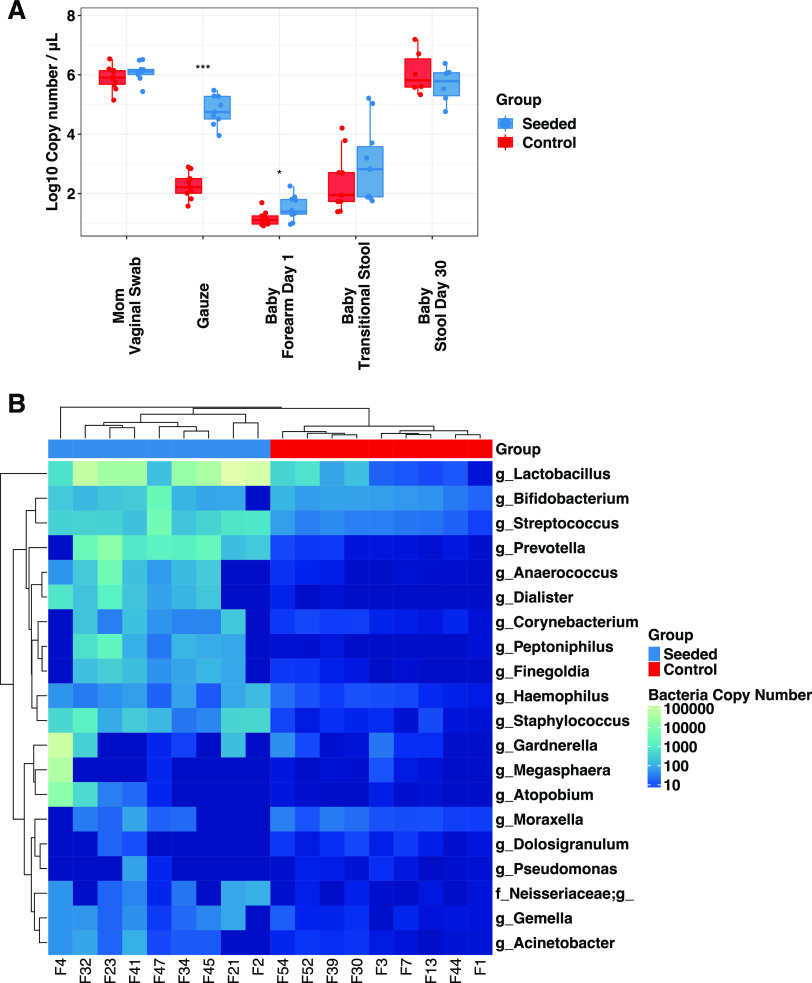
Differences in bacterial DNA load and composition between treatment groups at differing body sites, collection types, and times. (A) Bacterial DNA load in maternal vaginal swabs (both inoculated with vaginal fluids), gauze (control gauze not inoculated with vaginal fluids), infant skin (forearm), and infant stool. (B) Bacterial load of the 20 most abundant genera in gauzes from the two groups. Samples and taxa are ordered by unsupervised hierarchical clustering based on bacteria load. Bacterial load was estimated by 16S rRNA gene copy number based on qPCR using 16S rRNA gene universal primers. Wilcoxon signed-rank tests were performed for intergroup comparisons, with statistical significance indicated as follows: *, *P* < 0.05; ***, *P* < 0.001.

**FIG 2:**
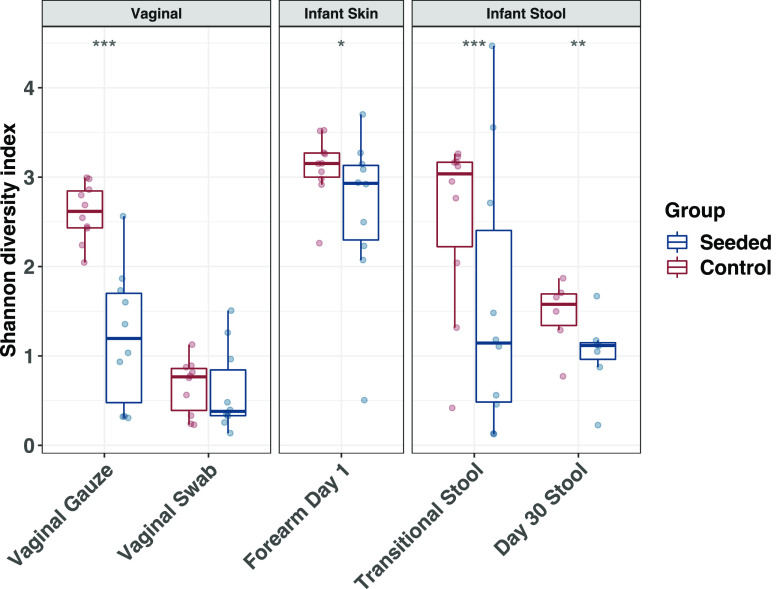
Shannon diversity index by treatment group at differing body sites, collection types, and times. Shannon diversity index values were estimated using the R package DivNet and assessed for significance using the function “betta” from the R package breakaway. Intergroup comparisons were considered statistically significant and were indicated as follows: *, *P* < 0.05; **, *P* < 0.01; ***, *P* < 0.001.

As expected, there were no differences in the maternal vaginal microbiota (prior to randomization) in terms of bacterial DNA load (measured by quantitative PCR [qPCR]) or alpha diversity, when comparing mothers from the vaginal-seeding versus control groups (Fig. 1A and 2A). After randomization, the bacterial load of the transfer gauze used to seed the neonate was higher in the vaginal-seeding group (average of 62,000 copies/μL) in relation to the control group (180 copies/μL; *P* = 5.1 × 10^−9^) (Fig. 1A). Relative to the control gauze, the vaginal gauze was enriched in *Lactobacillus* (19,000 copies/μL versus 33 copies/μL in the control; *P *= 0.0003) and *Bifidobacterium* (109 copies/μL versus 48 copies/μL; *P* = 0.0078) (Fig. 1B).

### Effect of vaginal seeding on the infant stool and skin microbiota.

Vaginal seeding significantly increased bacterial load in the skin (forearm; average, 32 copies/μL versus 14 copies/μL, in seeded and control babies, respectively; *P *= 0.033) but not in the transitional stool (*P *= 0.21) or day 30 stool (*P* = 0.32) (Fig. 1A).

Compared with the control, vaginal seeding caused a significant reduction in alpha diversity (Shannon index) in the skin at day 1 (Fig. 2B) and transitional stool of the neonates (Fig. 2C) (*P *= 0.047 for skin; *P *= 0.02 for stool), an effect that persisted in the infant stool microbiota by day 30 (Fig. 2C) (*P* = 0.01).

Treatment (seeding versus control) explained considerable variance in the beta diversity of the skin microbiota of day 1 neonates (*P = *0.02; 11.5% of variance explained by treatment arm) and to a slightly lesser degree, the transitional stool microbiota (*P* = 0.06; 10.0% of variance explained by treatment arm) (see Fig. S1A to C in the supplemental material). By day 30 after birth, the variance explained by treatment arm was considerably reduced for the stool microbiota (*P *= 0.62; only 3.1% of variance explained by treatment arm) (Fig. S1).

10.1128/mbio.00491-23.3 FIG S1 Principal-coordinate Analysis of infant microbiota beta diversity (weighted UniFrac) by treatment group. (A) Transitional stool. (B) Day 30 stool. (C) Day 1 skin. A permutational multivariate analysis of variance (PERMANOVA) test was performed, and statistical results were noted on the top of each panel. Download FIG S1, PDF file, 0.3 MB. Copyright © 2023 Mueller et al. 2023 Mueller et al. https://creativecommons.org/licenses/by/4.0/ This content is distributed under the terms of the Creative Commons Attribution 4.0 International license.

**FIG 3:**
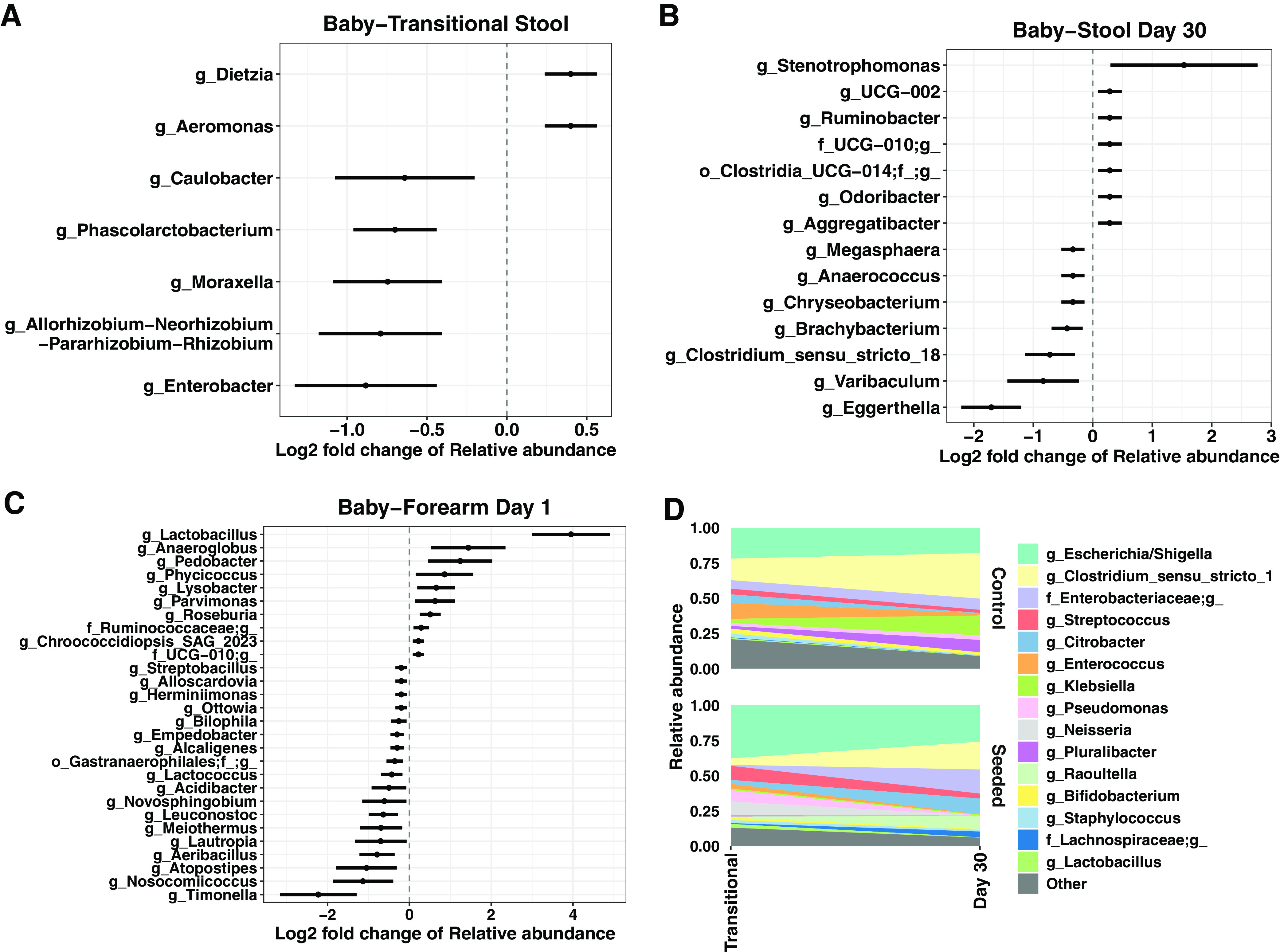
Log_2_ fold differences of ASV relative abundance by treatment group in transitional stool (A), day 30 stool (B), and day 1 skin (C). The dots represent the mean log_2_ fold change of relative abundance compared with the control group; the bars indicate the standard error of log_2_ fold change. All taxa were detected as significantly different between treatment group by ANCOM with default parameters and with a threshold of 0.9 (i.e., >90% comparisons indicate that the significant taxa have different relative abundance between groups). (D) Change in the relative abundance of top 15 taxa at the genus level between infant transitional stool and day 30 stool by treatment group.

Relative to the control, vaginal seeding altered the relative abundance of multiple bacterial amplicon sequence variants (ASVs), causing a decrease in the relative abundance of 5 ASVs in the transitional stool (detected by the analysis of compositions of microbiomes [ANCOM] method) (Fig. 3A), and an increase of 2 ASVs. By day 30, vaginally seeded babies had a decreased relative abundance of 7 ASVs and increased 7 ASVs (Fig. 3B). Interestingly, vaginal seeding decreased several potential pathobiont genera, such as Enterobacter, in transitional stool and *Clostridium sensu stricto* in day 30 stool (Fig. 3B and D). On the infant skin, vaginal seeding increased the relative abundance of 10 ASVs, including *Lactobacillus*, and lowered the abundance of 18 ASVs (Fig. 3C).

### Maternal sources of the infant microbiota.

**FIG 4:**
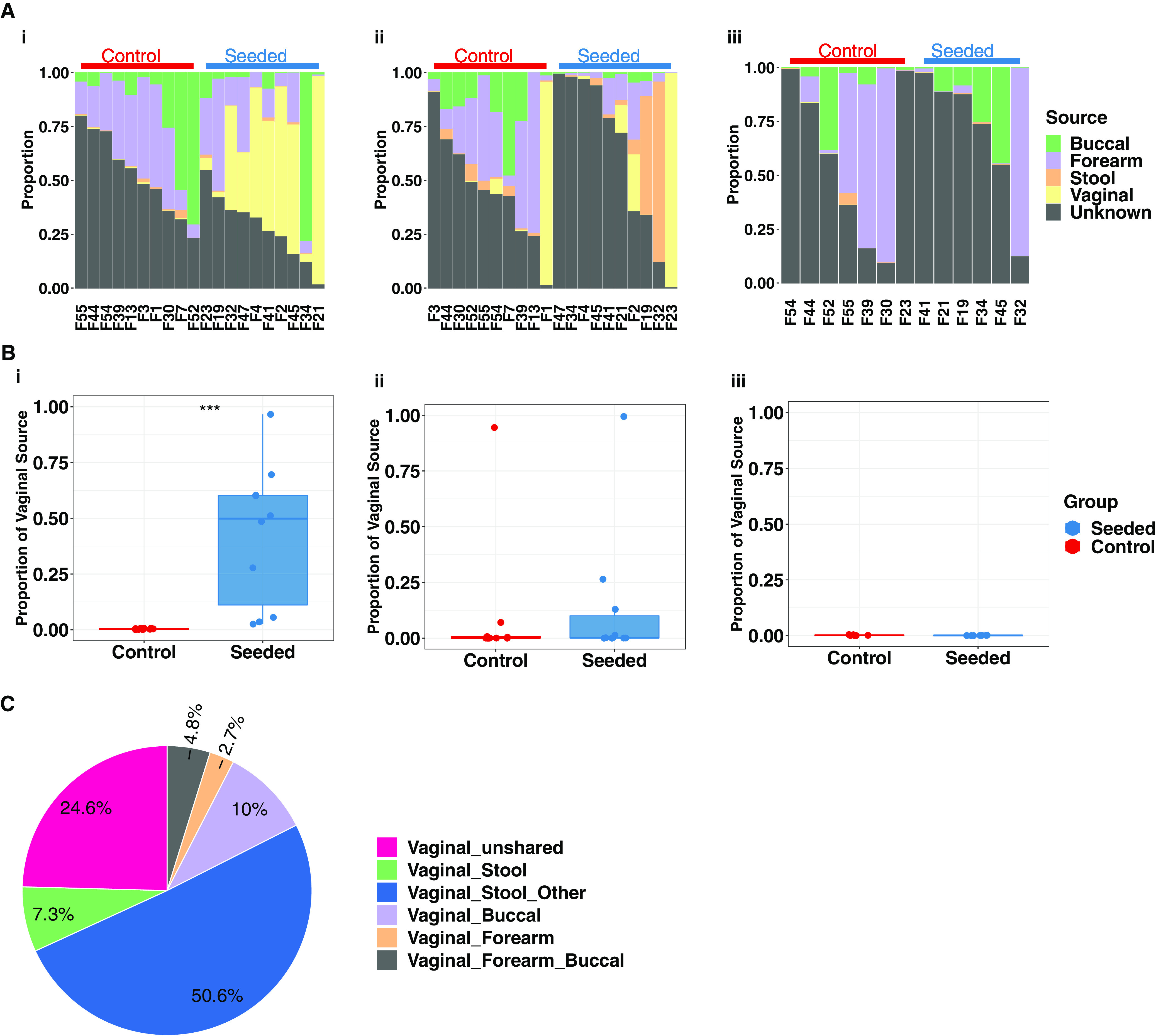
Proportion of maternal bacterial sources in infant and maternal body sites. (A) Contribution of maternal bacterial sources from different body sites to the microbiota of the infant skin (i), infant transitional stool (ii), and infant day 30 stool (iii). (B) Contribution of maternal vaginal bacteria (from vaginal swab) to the microbiota of the infant skin (i), infant transitional stool (ii), and infant day 30 stool (iii). (C) Bacterial sharing between vagina and other maternal body sites. Wilcoxon signed-rank tests were performed to compare source differences between groups, with statistical significance indicated as follows: *, *P* < 0.05; **, *P* < 0.01; ***, *P* < 0.001.

Source tracking analyses revealed a significantly higher proportion of maternal vaginal microbiota in the skin of 1-day old neonates, relative to control infants (adjusted *P* = 0.0001) (Fig. 4A and B). In the transitional stool, seeded babies showed lower maternal skin and oral microbiota sources than control infants (adjusted *P* = 0.009) (Fig. 4A). By day 30, there were no significant differences in maternal microbiota contributions in stool samples from the two treatment groups.

With respect to composition of maternal microbiota sources, the perinatal vaginal microbiota substantially overlapped with the microbiota in other maternal body sites (Fig. 4C), most notably with stool (57.9% of shared taxa; 168 shared ASVs) (see Fig. S2 in the supplemental material) but also with oral (10.0%) and skin (7.6% combined skin sources). This finding is consistent with the notion that the perinatal vaginal inoculum at the time of parturition contains pioneering bacterial taxa from multiple maternal body sites that can colonize multiple infant body sites.

10.1128/mbio.00491-23.4 FIG S2 Shared ASVs between vaginal swabs and maternal stool. Download FIG S2, PDF file, 0.07 MB. Copyright © 2023 Mueller et al. 2023 Mueller et al. https://creativecommons.org/licenses/by/4.0/ This content is distributed under the terms of the Creative Commons Attribution 4.0 International license.

## DISCUSSION

Our study is the first double-blind, randomized, placebo-controlled trial to determine whether vaginal seeding causes differential engraftment of maternal bacteria in the skin and stool of neonates. The study has important limitations, such as a small sample size and only 2 time points until 30 days of life. Despite these limitations, we observed significant effects of vaginal seeding on the neonatal microbiota. Vaginal seeding of C-section-delivered neonates increased mother-to-neonate microbial transfer and changed the neonatal microbiota in different body sites, urging further studies to determine whether these changes provide health benefits to the more than 1 million babies annually born by C-section in the United States (6).

The maturation of the stool microbiota in mammals progresses first with a postpartum reduction of the stool diversity—demonstrated in mice (24) and humans (23)—succeeded by a progressive increase in diversity later in infancy (7). We found in this study that alpha diversity is reduced in the transitional stool when the C-section neonate is vaginally seeded, which is consistent with the lower stool diversity in infants born vaginally in relation to C-section delivery (25). Breastfeeding, which was balanced between arms in our trial, has been shown to also lower stool alpha diversity in early infancy (26), by virtue of the strong selection of milk compounds in favor or a limited repertoire of beneficial organisms, such as *Bifidobacterium* and *Bacteroides* spp. (27). Thus, our results suggest that vaginal seeding abrogates the C-section effect of increasing stool diversity in neonates, similar to the effect of breastfeeding. We are currently following these infants until 3 years of age and will be able to assess the later maturation trajectory of the microbiota. Whether normalizing the low neonatal alpha diversity lowers C-section-associated diseases is unknown, but the hypothesis is supported by a longitudinal study that found higher alpha diversity in the stool of infants at 10 and 30 days of life was associated with higher BMI at 12 years of life (28).

C-section-born neonates have, in relation to their vaginally born counterparts, a higher proportion of potential pathobionts, such as from the *Enterobacteriaceae* family, and a lower proportion of beneficial microbes in the genera *Bacteroides* and *Bifidobacterium* (7, 15, 17–19). Consistently, in our study, vaginal seeding of C-section-delivered neonates decreased the abundance of *Enterobacteriaceae* (15, 19). However, there was no significant increase of *Bacteroides* and *Bifidobacterium*, with *Bacteroides* spp. having been reported to be higher among vaginally-seeded infants in some (22, 23) but not all (29, 30) studies of maternal-to-neonate vaginal microbial transfer after C-section. With respect to Bifidobacterium, in particular, breast milk is strongly bifidogenic in relation to formula (27), and it might be that breastfeeding—which was similar by treatment arm in our study—outweighed the effect of vaginal seeding. Other possible reasons for a lack of difference in these taxa could be the limited sample size for comparison at day 30, the first month of life being too early to observe differences (31), or that the gauze transfer conditions may not be optimal for preservation and transfer of these taxa.

Consistent with infants born vaginally showing stool bacterial communities resembling their mother’s vaginal microbiota and C-section-delivered infants showing skin-like microbiota (15–18), the control C-section-delivered neonates in the control arm of this study had more skin-like stool microbiota than the seeded group. Anomalies in the gut microbiota of C-section-delivered infants may disappear with time (14, 23), but even a transiently altered microbiome during a critical window of immune and metabolic programming may have long-term developmental consequences (5).

Our findings also support the growing recognition that microbiota from different maternal body sites are important for infant microbiome maturation (32). Indeed, the ideal microbiota source for a site in the infant body is likely the homologous maternal site, as shown by Korpela et al. (33) who demonstrated that fecal microbiome transplant from mother to child normalized the infant stool microbiota; however, they did not look at other neonatal body sites. Our results support the concept of “pluripotentiality” of the maternal perinatal vaginal microbiota (23), which furnished with bacteria from other body sites, can seed the homologous site in the baby. It is also worth noting that there are differences in human vaginal microbiota communities by race and ethnicity (34), which the small sample size of the current study could not capture well, and in the future, further studies are needed to characterize vaginal community state types (I, II, III, IV, or V) in relation to seeding efficiency and outcomes.

In conclusion, vaginal seeding caused changes in the neonatal skin and stool microbiota, leading to a pattern of reduced bacterial diversity that is characteristic of the microbiota of vaginally delivered and breastfed infants. There is now a critical need to evaluate the health benefits and safety of vaginal seeding in large randomized controlled trials.

## MATERIALS AND METHODS

### Study subjects.

We performed this institutional review board (IRB)-approved study (WCG IRB number 1300043) at the Inova Health System in Northern Virginia under the US Food and Drug Administration (FDA) Investigational New Drug Application (IND) number 18076 (with an IND required by the FDA). We recruited pregnant women who were scheduled for an elective C-section at ≥37 weeks gestation and obtained written informed consent. We applied stringent inclusion and exclusion criteria to ensure the least risk of maternal-to-infant transmission of infection as discussed with the FDA and the exclusion of maternal health conditions associated with dysbiosis of the vaginal microbiome. For full details of inclusion and exclusion criteria, see supplemental data and https://clinicaltrials.gov/ct2/show/NCT03298334.

### Study procedure: randomization and vaginal seeding.

On the day of the scheduled C-section, a team member blind to treatments inserted a gauze moistened with sterile saline into the mother’s vagina. The gauze was incubated in the vagina for approximately 1 h and then removed and placed into a lidded sterile container prior to the mother receiving perioperative antibiotics (with the exception of 2 mothers who were penicillin allergic and received clindamycin and gentamicin when the gauze was still in the vagina due to the longer antibiotic infusion time) and kept at room temperature. Shortly after the insertion of the vaginal gauze, a control gauze was also made by identical preparation of the gauze (i.e., folding of the gauze and moistening with sterile saline following the same protocol as the vaginal gauze) but without insertion into the mother’s vagina. The control gauze was also placed in a separate lidded sterile container and kept at room temperature. A team member not blind to the treatments then randomized the mother to either the vaginal seeding arm or the control arm. Randomization occurred in a 1:1 ratio and was also stratified to the following 3 prepregnancy BMI strata: normal weight, overweight, and obese.

In the operating room, after C-section delivery of the infant and prior to the infant having skin-skin contact with its mother, the infant was wiped down with the vaginal seeding gauze or the control gauze by a team member who was blind to the treatments. The gauze was wiped first over the infant’s mouth, followed by the face and the rest of the body in a standardized manner as described previously, typically just after the 1-min Apgar score was taken (22, 23).The vaginal gauze used to wipe down the infant was retained and stored at −80°C until processing.

### Data and samples.

We collected detailed data on mothers, including demographics, medical history, anthropometrics, and medication use, including antibiotics during pregnancy. Data collected for the infants included demographics, method of first feeding and subsequent feedings, medication use, and adverse events. We collected and managed study data using the REDCap electronic data capture tools hosted at the Inova Health System.

For this study, maternal samples collected and analyzed were as follows: (i) prenatal maternal stool from after 35 weeks gestation, (ii) vaginal swab collected just prior to the vaginal gauze insertion before randomization, (iii) gauze used for swabbing the neonate, (iv) skin swab from the forearm obtained the day after delivery, and (iv) buccal swab obtained the day after delivery. Infant samples collected and analyzed were as follows: (i) transitional stool sample at day of life 2 to 3, (ii) stool sample around day 30 of life (mean, 34.9 days [SD 3.73]), and (iii) skin swab from the forearm (using a dry sterile swab rolled over forearm skin area of approximately 2 by 2 inches) obtained the day after delivery before the infant’s first bath. We stored all samples at −80°C until analysis.

### DNA extraction and sequencing.

We extracted microbial DNA using the Qiagen DNeasy PowerSoil HTP 96 kit (Hilden, Germany) following the manufacturer’s instructions. For 16S rRNA gene sequencing, we amplified the V4 region of 16S rRNA genes using the 515F/806R primers according to the Earth Microbiome Project protocol (35). We sequenced prepared amplicon libraries at Genewiz, LLC. (South Plainfield, NJ). Positive controls (ZymoBiomics microbial community standard, catalog number D6300; Zymo Research, CA) and negative controls (empty extraction tubes) were included.

### Determination of bacterial DNA load.

We determined bacterial copy number by qPCR using a Quantstudio 3 system (Thermo Fisher, Waltham, MA) with universal 16S gene primers (338F, ACTCCTACGGGAGGCAGCAG; and 518R, ATTACCGCGGCTGCTGG) (36). We conducted the qPCRs using a Quantinova SYBR green PCR kit (Qiagen, Hilden, Germany) following to the manufacturer’s instructions; the final volume of extracted DNA was 100 μL, and we used 1 μL of extracted DNA as the template. We used Escherichia coli genomic DNA with known copy numbers as the standard.

### Metadata analysis.

All analyses were conducted by the statistical team in a blind manner. Select members of the statistical team were unblinded only after the final analysis of the data. Descriptive statistics (median with interquartile range and percentages) were presented on demographic and clinical characteristics for mothers and infants according to treatment arm.

### Microbiota analysis.

The bioinformatics team conducted all microbiota analyses in a blind manner, and select members were unblinded only after the final analysis.

### 16S rRNA gene sequencing processing.

We quality controlled dereplicated 16S sequences without primers and denoised them into amplicon sequence variants (ASVs) using the R package DADA2 (v1.20.0) using the pooled settings for denoising and chimera removal (37, 38). We trimmed the first and last reads from the forward sequences and the first and last reads from reverse sequences after evaluating the distribution of sequence quality scores. We used the R package “decontam” to address any possible environmental contamination using the DNA quantitation method aided by comparisons to negative controls for each sample type. On average, 82% of sequences were maintained from raw files to final QCed reads. We assigned taxonomy using the “assignTaxonomy” and “addSpecies” functions from DADA2 using the SILVA v138 small subunit rRNA reference files (39–41). We generated a generalized time-reversible with gamma rate variation (GTR) maximum likelihood phylogenetic tree from the final ASVs and rooted it at the midpoint using the R packages DECIPHER (v2.20.0), phangorn (v2.7.1), and ape (v5.5) according to the specifications recommended by an established workflow (38). We used the R package phyloseq (v1.36.0) to join the ASVs, phylogenetic tree, taxonomy, and sample metadata into a single phyloseq object for analysis. We rarefied samples to a depth of 4,000 reads for analyses that relied on rarefied data (see below).

### Estimation of microbial beta diversity.

We calculated weighted UniFrac distances to obtain the pairwise beta diversity, which we further evaluated by the adonis2 function using the R package vegan to test the significance with 999 permutations (42). We performed principal-coordinate analysis (PCoA) of weighted UniFrac distances to reduce the dimensionality.

### Estimation of microbial alpha diversity.

We used the R package DivNet to estimate Shannon diversity using ecological network regression models. We employed the function “betta” (43) from the R package breakaway to test for differences in Shannon diversity across groups.

### Source tracking of microbiota.

To estimate the sources of the infant microbiota at different body sites, we performed bacterial source tracking using FEAST (44). Sources for this analysis were samples from the corresponding mother’s vagina, stool, buccal source, and forearm. We designated infant’s stool and forearm samples as sink samples (target microbial community). We performed the analysis on rarified count tables with 4,000 reads per sample with 1,000 expectation-maximization (EM) iterations.

### Differential abundance analysis.

We used ANCOM to search for differentiated bacterial taxa by randomization group (45). We further analyzed significant taxa using a linear model to determine the enrichment direction and fold change.

### Data availability.

Sequencing reads were deposited in the Sequence Read Archive (SRA) under PRJNA954027 and are publicly available as of the date of publication.

10.1128/mbio.00491-23.2 DATA SET S1 Inclusion and exclusion criteria for the clinical trial. Download Data Set S1, PDF file, 0.07 MB. Copyright © 2023 Mueller et al. 2023 Mueller et al. https://creativecommons.org/licenses/by/4.0/ This content is distributed under the terms of the Creative Commons Attribution 4.0 International license.
