# Supplementary material for: Maternal Bacterial Engraftment in Multiple Body Sites of Cesarean Section Born Neonates after Vaginal Seeding—a Randomized Controlled Trial
Source: mBio. 2023 Apr 19;14(3):e00491-23. doi: 10.1128/mbio.00491-23 (PMC10294643; doi:10.1128/mbio.00491-23)
Supplement: TABLE S1 [file mbio.00491-23-s0001.pdf]

Supplemental Table 1. Baseline characteristics of randomized infants and mothers.

	Seeded (n=10)	Control (n=10)
**Infant characteristics**		
Male infant gender	6 (60.0%)	6 (60.0%)
Gestational age (weeks)	38.5 [37.2, 39.0]	39.0 [39.0, 39.0]
Birth weight (kg)	3.1 [3.0, 3.2]	3.4 [3.1, 3.6]
Infant antibiotics by 7 days	0 (0%)	0 (0%)
Infant antibiotics by 30 days	0 (0%)	0 (0%)[a]
Method of first feed		
Breast	8 (80%)	10 (100%)
Formula	1 (10%)	0 (0%)
Both	1 (10%)	0 (0%)
Infant feeding at 7 days		
Breastmilk only (%)	5 (50%)	7 (70%)
Formula and breastmilk (%)	4 (40%)	3 (30%)
Formula only (%)	1 (10%)	0 (0%)
**Maternal characteristics**		
Maternal age (years)	34.0 [32.0, 37.2]	33.0 [30.5, 34.8]
Maternal race		
White	5 (50%)	9 (90%)
Asian	3 (30%)	1 (10%)
Other	1 (10%)	0 (0%)
Declined to provide	1 (10%)	0 (0%)
Maternal pre-pregnancy BMI (kg/m^2)	24.3 [21.7, 28.0]	26.6 [22.2, 29.7]
Antibiotics during pregnancy	3 (30%)	1 (10%)
Gestational diabetes	1 (10%)	0 (0%)
Antibiotics during gauze because of Penicillin allergy	2 (20%)	0 (0%)

Data presented as median [IQR] or n (%). [a]2 infants missing this information
