# Supplementary figures and images for: Maternal Bacterial Engraftment in Multiple Body Sites of Cesarean Section Born Neonates after Vaginal Seeding—a Randomized Controlled Trial
Source: mBio. 2023 Apr 19;14(3):e00491-23. doi: 10.1128/mbio.00491-23 (PMC10294643; doi:10.1128/mbio.00491-23)

**A****Baby-Transitional Stool**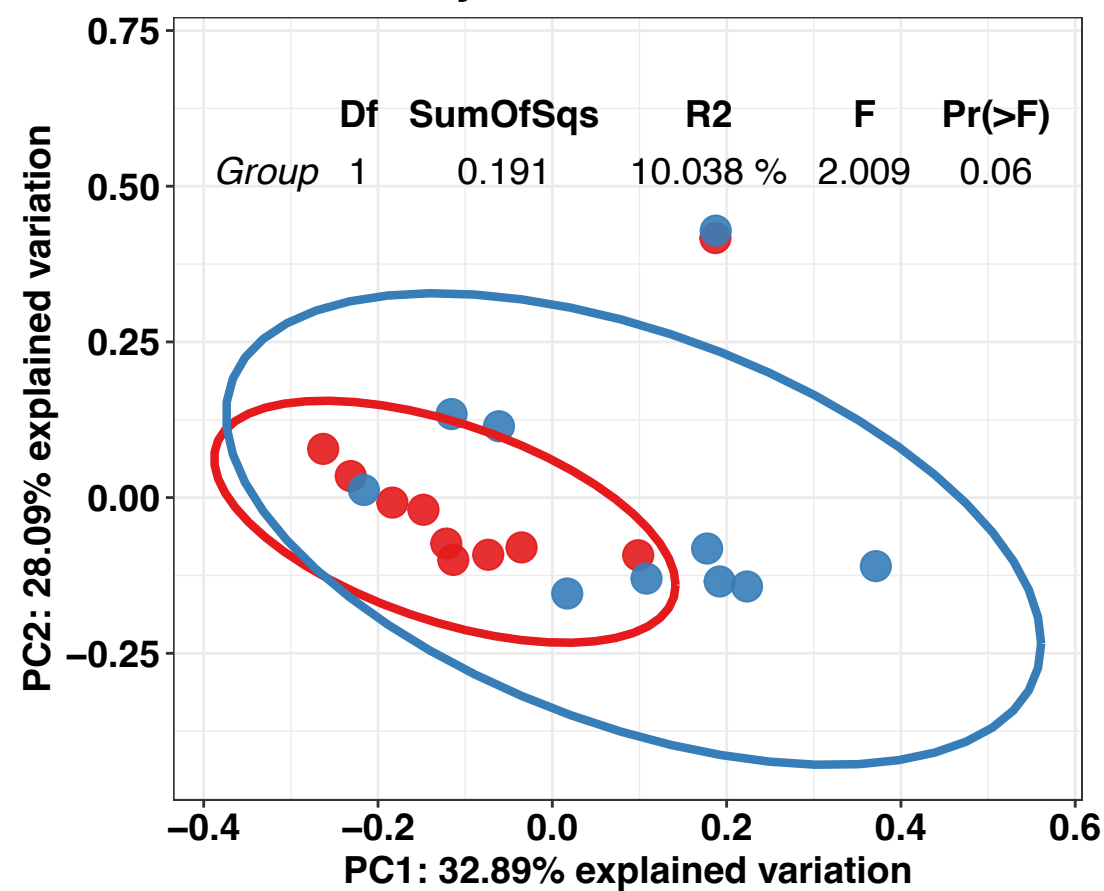**B****Baby-Stool Day 30**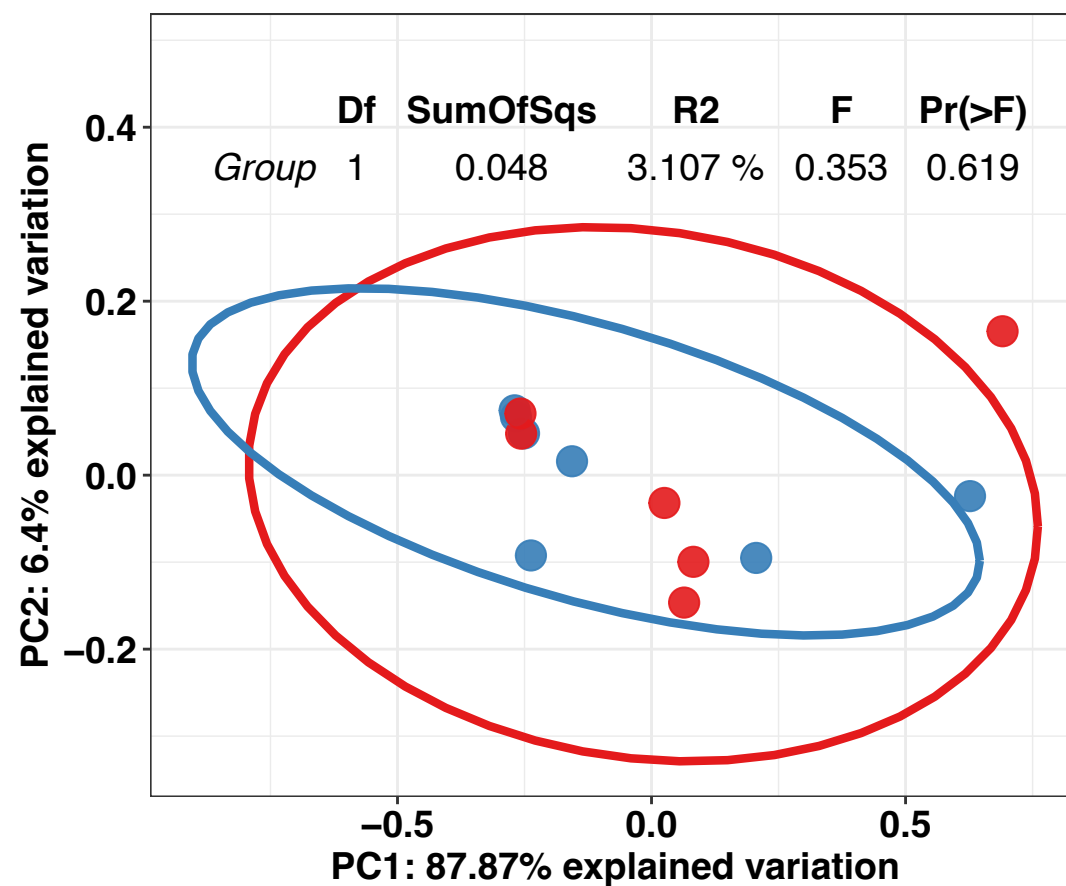**C****Baby-Forearm Day 1**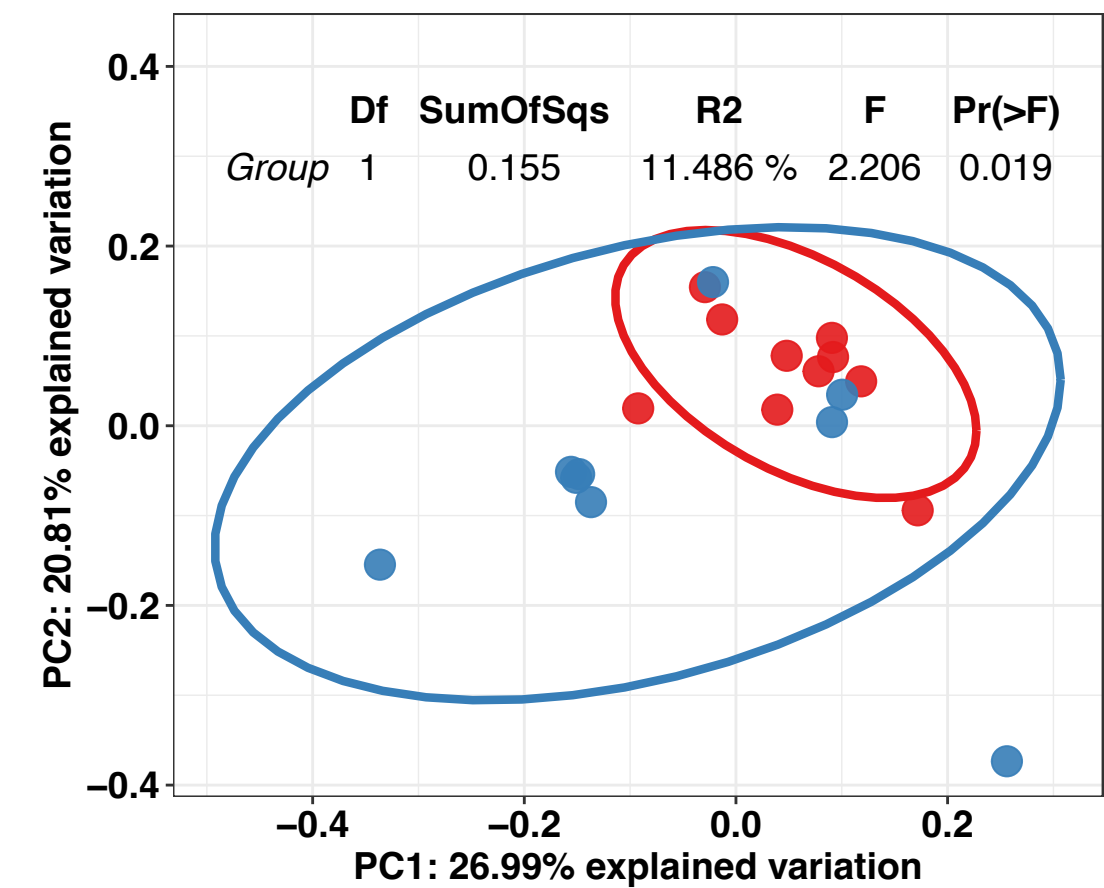**Group**

● Seeded

● Control

Supplement: FIG S1 [file mbio.00491-23-s0003.pdf]

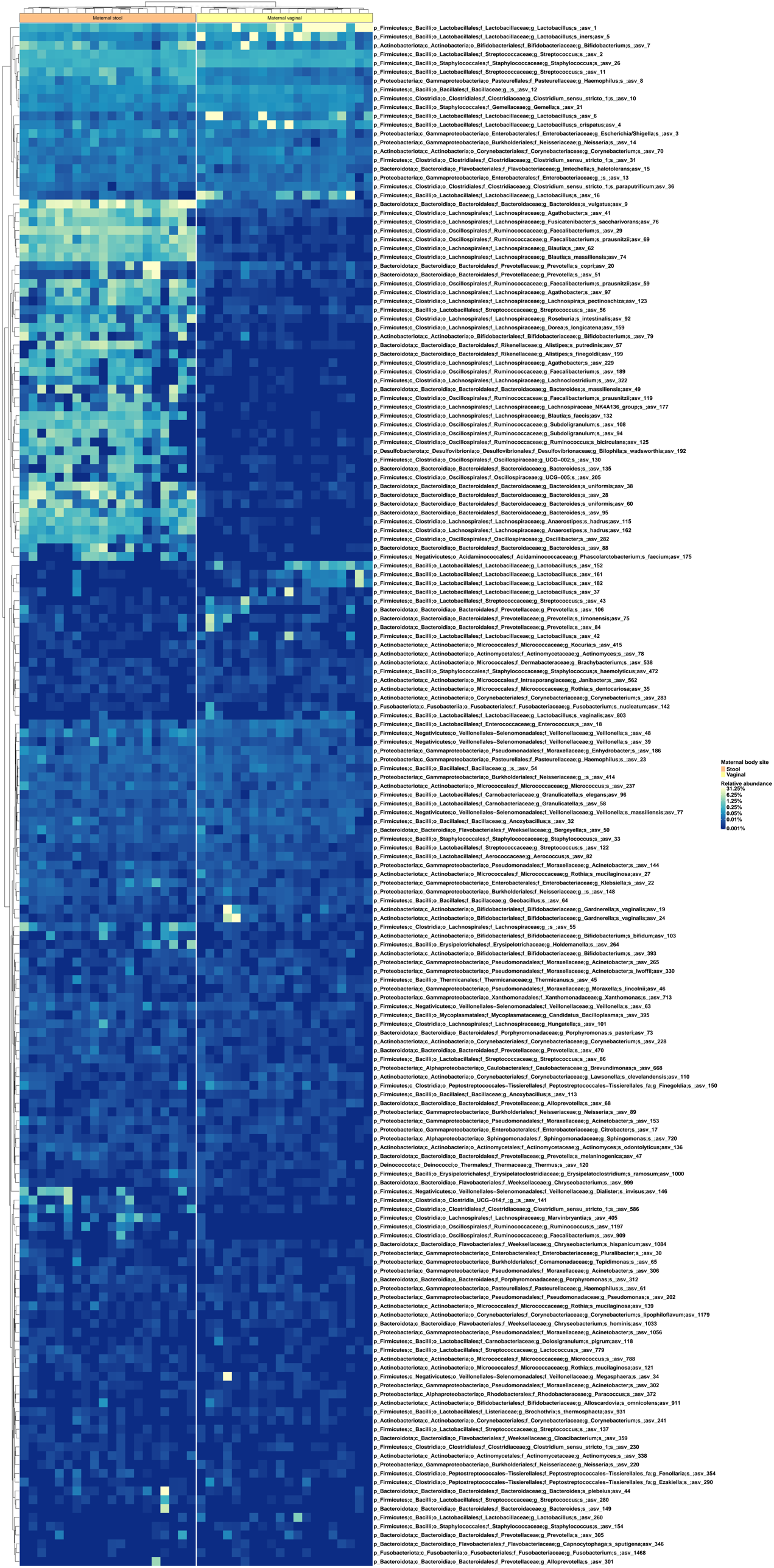

Supplement: FIG S2 [file mbio.00491-23-s0004.pdf]
