# Supplementary material for: Maternal Bacterial Engraftment in Multiple Body Sites of Cesarean Section Born Neonates after Vaginal Seeding—a Randomized Controlled Trial
Source: mBio. 2023 Apr 19;14(3):e00491-23. doi: 10.1128/mbio.00491-23 (PMC10294643; doi:10.1128/mbio.00491-23)
Supplement: DATA SET S1 [file mbio.00491-23-s0002.pdf]

Supplemental Data: Inclusion and exclusion criteria for the clinical trial.

Inclusion Criteria for Mother:

- Scheduled for cesarean delivery at ≥ 37 weeks
- Pregnant with single fetus, in good general health, age 18 years or older
- Negative maternal testing for infections transmitted through vaginal and/or other body fluids performed as standard of care tests in early pregnancy
- Negative testing for Group B strep at 35-37 weeks gestation
- Vaginal pH ≤ 4.5 indicative of Lactobacillus-dominated vaginal microbiota
- No maternal or fetal complications that may inhibit the ability to perform microbiome restoration per protocol
- English or Spanish speaking
- Negative maternal testing for Gonorrhea, Chlamydia, Hepatitis B, Hepatitis C, Syphilis, and HIV at 35 weeks gestation or later
- Women aged 18-29 years must have a normal Pap test within 3 years
- Women aged 30-65 years must have a normal Pap test and an HPV test (co-testing) within 5 years or FDA-approved primary hrHPV testing alone within 5 years or a normal Pap test alone within 3 years
- Negative maternal testing for SARS-CoV-2 for the delivery admission performed as standard of care test at the Inova Health System.

Inclusion Criteria for Infant:

- Infant condition after delivery requires no more than standard neonatal resuscitation* or is otherwise medically unable to receive the full VMT procedure

[*] Standard neonatal resuscitation may include: tactile stimulation, bulb suction, oxygen without positive pressure, or drying

Exclusion Criteria for Mother:

- Delivery at a hospital other than Inova Health System
- Cesarean delivery scheduled for active infection that would have interfered with vaginal delivery such as genital herpetic lesions
- Rupture of membranes prior to scheduled cesarean delivery
- Bacterial vaginosis within 30 days of cesarean delivery
- Symptomatic urinary tract infection within 30 days of cesarean delivery
- Antibiotic therapy within 30 days of cesarean delivery (exclusive of medication use for prophylaxis at the time of surgery)
- Symptoms on admission suggesting Chorioamnionitis, e.g. maternal fever, fundal tenderness
- Symptoms on delivery admission of possible vaginal infection such as genital herpetic lesions
- History of genital HSV
- History positive testing for Group B strep infection

- History of a child with a diagnosis of Group B strep sepsis
- Pregnancy a result of donor egg or surrogacy
- Preexisting history of Type I or Type II Diabetes
- Maternal history of documented genital HPV infection, positive HPV testing or genital warts on physician examination
- Positive maternal testing for SARS-CoV-2 within 30 days of delivery or symptoms on admission suggesting potential Covid-19 infection
